# Humoral and cellular immune responses to CoronaVac up to one year after vaccination

**DOI:** 10.3389/fimmu.2022.1032411

**Published:** 2022-10-21

**Authors:** Priscilla Ramos Costa, Carolina Argondizo Correia, Mariana Prado Marmorato, Juliana Zanatta de Carvalho Dias, Mateus Vailant Thomazella, Amanda Cabral da Silva, Ana Carolina Soares de Oliveira, Arianne Fagotti Gusmão, Lilian Ferrari, Angela Carvalho Freitas, Elizabeth González Patiño, Alba Grifoni, Daniela Weiskopf, Alessandro Sette, Rami Scharf, Esper Georges Kallás, Cássia Gisele Terrassani Silveira

**Affiliations:** ^1^ Medical Investigation Laboratory 60 (LIM-60), School of Medicine, University of São Paulo, São Paulo, Brazil; ^2^ Department of Infectious and Parasitic Diseases, Clinicas Hospital, School of Medicine, University of São Paulo, São Paulo, Brazil; ^3^ Center for Clinical Trials and Pharmacovigilance Butantan Institute, São Paulo, Brazil; ^4^ Center for Infectious Disease and Vaccine Research, La Jolla Institute for Immunology, San Diego, CA, United States; ^5^ Department of Medicine, Division of Infectious Diseases and Global Public Health, University of California San Diego, San Diego, CA, United States; ^6^ PATH, Washington, DC, United States

**Keywords:** CoronaVac, vaccination, cellular response, humoral response, immune memory

## Abstract

Coronavac is a widely used SARS-CoV-2 inactivated vaccine, but its long-term immune response assessment is still lacking. We evaluated SARS-CoV-2-specific immune responses, including T cell activation markers, antigen-specific cytokine production and antibody response following vaccination in 53 adult and elderly individuals participating in a phase 3 clinical trial. Activated follicular helper T (Tfh), non-Tfh and memory CD4^+^ T cells were detected in almost all subjects early after the first vaccine dose. Activated memory CD4^+^ T cells were predominantly of central and effector memory T cell phenotypes and were sustained for at least 6 months. We also detected a balanced Th1-, Th2- and Th17/Th22-type cytokine production that was associated with response over time, together with particular cytokine profile linked to poor responses in older vaccinees. SARS-CoV-2-specific IgG levels peaked 14 days after the second dose and were mostly stable over one year. CoronaVac was able to induce a potent and durable antiviral antigen-specific cellular response and the cytokine profiles related to the response over time and impacted by the senescence were defined.

## Introduction

Protective and long-lasting immune responses induced by viral infections and vaccines are usually formed by a combination of humoral and cellular immunity. The presence of both SARS-CoV-2-specific cells and circulating antibodies after infection or vaccination are the most likely candidates to serve as correlates of protection against COVID-19 and disease severity. High frequency of circulating SARS-CoV-2-specific CD4^+^ and CD8^+^ T cells in patients who recovered from COVID-19, as well as the presence of memory T cells in the convalescent phase have been extensively described in the literature and are suggested to play a key role in controlling SARS-CoV-2 initial infection, protecting from re-infection and disease progression (1–3).

CoronaVac, a whole SARS-CoV-2-inactivated vaccine administered to over 2 billion people (4), has an excellent safety profile and is effective against symptomatic SARS-CoV-2 infections and is highly protective against moderate and severe COVID-19 in clinical trials performed in Brazil (5,6) and other countries (7–9). Previous studies showed that two doses can elicit neutralizing antibodies (6) and described short-term cellular responses following vaccination (10). The PROFISCOV phase 3 clinical trial of CoronaVac vaccine in Brazil started on July 21, 2020 and enrolled 12,396 highly-exposed healthcare professionals who received the two-dose vaccine or placebo (11). A sub-cohort of adult and elderly individuals was selected for a prospective study to describe the vaccine-induced cellular and humoral immune responses in the peripheral blood, including the assessment of SARS-CoV-2 specific T cell responses, T cell-related cytokine and chemokine profiles, and plasma binding antibody levels up to one year after vaccination.

## Materials and methods

### PROFISCOV phase 3 clinical trial

To assess the safety and efficacy of the CoronaVac vaccine in Brazil, a randomized, double-blind, placebo-controlled phase 3 multicenter clinical trial was performed in healthy healthcare professionals on the frontline of COVID-19 pandemic. The trial was approved by the Brazilian National Research Ethics Council (CONEP), CAAE 34634620.1.1001.0068, the Brazilian National Regulatory Agency (ANVISA), CE 47/2020, and was registered in the ClinicalTrials.gov platform (NCT04456595). The full protocol of the clinical trial has been published previously by Palacios et al. (11). All participants provided written informed consent.

CoronaVac (Sinovac Life Sciences, Beijing, China) is an inactivated vaccine derived from the CN02 strain of SARS-CoV-2 grown in Vero cells. Production methods and its full composition has been published by Gao et al. (12). The placebo group received aluminum hydroxide adjuvant with no virus. Vaccine and placebo were provided in a ready-to-use syringe and administered intramuscularly following the two-dose schedule of 0 and 14 days (11).

12,396 initial participants were recruited in the Brazilian phase 3 clinical trial, with ages ranging from 18 to over 60. From those, 653 were included in the São Paulo site. The first 120 had blood collection and peripheral blood mononuclear cells (PBMC) isolation for a long-term follow-up and long-term assessment of their cellular and humoral responses.

### Study design and participants

After the breaking of the participants’ blinding code, the 120 first individuals (60 vaccinees and 60 placebos) were filtered based on vaccination status, age and specimen availability to compose the cohort used to assess cellular and humoral response after immunization with CoronaVac. The final cohort was primarily composed of 29 vaccinees and 4 placebos ranging from 18 to 59 years, and 24 vaccinees and 4 placebos with ages over 60 years (with a total of 53 vaccinees). Seven vaccinated volunteers were excluded from further analysis due to lack of follow-up or positive COVID-19 diagnosis right after the first vaccine dose. Placebos were only evaluated before vaccination and there were no infections reported in this group at this time point.

Blood samples were collected before vaccination (0d), 14 days after the first dose (14d, collected just before the second dose) and in several other time points counted as days after the first vaccine dose (30d, 60d, 90d, 120d, 180d, 360d).

In order to quantify the response magnitude achieved after vaccination, 17 SARS-CoV-2 infected, age-paired individuals were selected as positive controls (eight non-vaccinated, hospitalized patients due to moderate COVID-19 symptoms and nine non-vaccinated individuals with mild symptoms that did not require hospitalization). Blood samples from these hospitalized patients were part of the SARS-CoV-2 neutralizing antibody investigation research project approved by CONEP, CAAE 34634620.1.1001.0068, and the non-hospitalized patients were infected placebos from the CoronaVac clinical trial. Non-hospitalized patients were only included in some assays due to the lack of PBMC samples. The demographics data of the included individuals are described in **Table 1**. In each figure representing results the number of analyzed individuals and the respective time points are described in the legends.

**Table 1:** Demographic data of the study’s participants.

Groups	Age	Sex	Days between vaccine doses	Days of symptoms onset	COVID-19 diagnosis
Placebo (n=8)	54 (36-64)	62,5% F	–	–	–
		37,5% M			
**Vaccinees**					
18-59 (n=29)	36 (31-42)	55,2% F	15 (14-18)	–	–
		44,8% M			
≥60 (n=24)	67 (63-70)	33,3% F	16 (14-21)	–	–
		66,7% M			
**COVID-19 cases**					
Hospitalized (n=8)	53 (43-62)	12,5% F	–	19 (17-22)	75% PCR
		87,5% M			25% Serology
Non-hospitalized (n=9)	40 (33-44)	55,6% F	–	46 (34-65)	100% PCR
		44,4% M			

### Sample processing

Blood was collected in Vacutainer^®^ ACD tubes (BD Biosciences, CA, USA). They were centrifuged at 10 min for 1800rpm and plasma was collected in a 15 mL tube. Plasma was centrifuged at 10 min for 2800rpm for the precipitation of debris, and then aliquoted at 1 mL tubes and preserved in -80°C freezer for further use.

The blood phase was diluted 1:1 in Hank’s Balanced Salt Solution (HBSS 1X) (Gibco, MA, USA) and then slowly transferred to tubes with 1:3 gradient density Ficoll-Paque™ PLUS (Marlborough, MA, USA), to separate PBMCs. The diluted blood was centrifuged at 30 min for 2300 rpm with no acceleration/deceleration. After centrifugation, the PBMCs cloud was collected, diluted in HBSS and centrifuged at 10 min for 1700 rpm. The supernatant was discarded, the cell pellet was resuspended and counted in Countess™ (Thermo Scientific, MA, USA), diluted 1:1 with Trypan Blue Stain (Thermo Scientific, MA, USA). After counting cells were centrifuged again at the same condition and resuspended in freezing media, composed by 10% dimethyl sulfoxide (DMSO, Sigma-Aldrich, Darmstadt, Germany) and 90% fetal bovine serum (Gibco, MA, USA). Cells were frozen at a maximum of 10x10^6^ cells/mL at liquid nitrogen until further assays.

### Activation-induced markers T cell assay

Evaluation of cellular parameters through activation-induced markers (AIM) assay included assessment of SARS-CoV-2-specific CD8^+^ and CD4^+^ T cell responses using virus-specific peptide megapools (MP) developed by Grifoni and colleagues (1), designed to overlap the Wuhan/WH04/2020 SARS-CoV-2 ORFeome. To surpass sensitivity limitations of cytokine-based assays, the AIM assay of CD4^+^ and CD8^+^ T cells was performed, which defines antigen specificity based on positive regulation of surface markers induced by T cell receptor (TCR) stimulation, instead of cytokine production. For this purpose, PBMCs from vaccinees and placebos were stimulated with MPs comprehending different portions of SARS-CoV-2, in order to quantify and determine the subsets of antigen-specific CD4^+^ and CD8^+^ T cells stimulated after the vaccine.

PBMCs were thawed at 37°C and diluted in RPMI 1640 media (Thermo Scientific, MA, USA) supplemented with 5% human serum (Sigma-Aldrich, Darmstadt, Germany), 2 mM L-glutamine (Gibco, MA, USA), 1 mM penicillin/streptomycin (Gibco, MA, USA), 10 mM HEPES solution (Gibco, MA, USA), 1mM sodium pyruvate (Gibco, MA, USA) and 55 mM β-mercaptoethanol (Gibco, MA, USA) (hereafter referred as HR5). Cells were centrifuged at 1500 rpm for 10 min, resuspended in HR5 containing 50 U/mL benzonase (Merck, Darmstadt, Germany) and incubated at 37°C and 5% CO2 to remove DNA/RNA aggregates. After incubation, cells were counted, centrifuged at 1500 rpm for 10 min and seeded at a density of 1,5x10^6^ cells per well, in U-bottom 96-well plates.

Cells were incubated with 0,5 μg/mL anti-CD40 antibody (Miltenyi Biotec, NRW, Germany) for 15 min at 37°C and 5% CO2 and then stimulated for 24h at 37°C and 5% CO2 in the presence of specific MPs (1 μg/mL), phytohemagglutinin (PHA, Sigma-Aldrich, Darmstadt, Germany) (10 μL/mL) as a positive control or dimethyl sulfoxide (DMSO, Sigma-Aldrich, Darmstadt, Germany) (0,1%) as a negative control, all diluted in HR5. MPs were developed and kindly donated by Dr. Alessandro Sette’s laboratory (Center for Infectious Disease and Vaccine Research; La Jolla Institute for Immunology, USA) (1) and comprehend SARS-CoV-2-specific epitopes specified as CD4-R (remaining non-Spike protein), CD4-S (Spike protein) and CD8-A and CD8-B (viral epitopes compatible with HLA-A and HLA-B, respectively).

After a 24-hours MPs incubation, plates were centrifuged at 1800 rpm for 2 min and the supernatant was collected in a new plate and kept at -80°C freezer for further analysis. Cell pellets were resuspended, transferred to V-bottom 96-well plates and washed with MACS buffer (5 mg/mL bovine serum albumin (BSA, Sigma-Aldrich, Darmstadt, Germany) and 2 mM ethylenediamine tetraacetic acid (EDTA, Sigma-Aldrich, Darmstadt, Germany) diluted in phosphate-buffered saline 1X (PBS, LGC Biotecnologia, SP, Brazil)) twice, then stained with specific antibodies mix containing characterization and activation markers (**Table S1**) for 20 min, at 4°C in the dark (13). After staining, cells were washed twice at the same conditions, fixed with paraformaldehyde (PFA, Sigma-Aldrich, Darmstadt, Germany), diluted 1:10 in PBS 1X, for 10 min at room temperature in the dark, centrifuged at 2000 rpm for 5 min, resuspended in PBS 1X and acquired at the BD LSRFortessa™ X-20 Cell Analyzer (BD Biosciences, CA, USA).

The quality control from samples acquired in the flow cytometer was performed by the analysis of the Fluorescence Minus One (FMO) and compensation adjustments through beads. Acquisition was performed with the BD FACSDiva™ Software v6.0 and FlowJo™ v10.8 was used for data analysis (both from BD Biosciences, CA, USA). T cell response values after viral peptide stimulus were obtained from the subtraction of the peptide-stimulated conditions from the DMSO condition. To generate data of total CD4^+^ and CD8^+^ T cells, separate responses for CD4-R and CD4-S and a sum of both were performed, as well for CD8-A and CD8-B.

### Cytokine and chemokine quantification

Cytokines and chemokines were assessed in the supernatant derived from the AIM T CD4^+^ cell assay after the 24h incubation and prior to cell staining. The following MSD^Ⓡ^ (Meso Scale Discovery, MD, USA) human kits were utilized: Chemokine Panel 1 (K15047G), Cytokine Panel 1 (K15050G), Proinflammatory Panel 1 (K15049G) and Th17 Panel 1 (K15085D). All biomarkers from the corresponding panels are described in **Table S2**, as well as the detection range of each assay. All kits were performed following manufacturer’s instructions. To generate data of CD4^+^ cells supernatant biomarkers quantification, we subtracted the CD4-R and S MPs from DMSO and summed both CD4-R and CD4-S supernatant responses for each biomarker.

### Binding antibody assay

Plasma samples were tested for quantitative IgG bAbs against nine SARS-CoV-2 antigens: Spike (S), RBD and Nucleotide (N) from the Wuhan/WH04/2020 strain and S and RBD from the VOCs Alpha (B.1.1.7), Beta (B.1.351) and Gamma (P.1). An electrochemiluminescence multiplex serology assay (V-PLEX SARS-CoV-2 Panel 7 IgG Kit, K15437U, MesoScale Discovery (MSD), MD, USA) was used following manufacturer’s instructions. Plasma samples were heat inactivated at 56°C for 45 minutes and used at a 1:1000 dilution.

Raw data was generated by Methodical Mind software (version 1.0.37; MSD) and analyzed with Discovery Workbench software (version 4.0; MSD). Antibody concentrations were calculated based on the 8-point calibration curve, specific for each one of the nine antigens, and reported as arbitrary units (AU/mL) adjusted by the dilution factor used in the assay.

### Heatmap, correlation plots and data visualization

Heatmap and correlation plots were created under R (version 4.1.2) in Rstudio Cloud (version 2022.02.1). Data was standardized to a z-score using the function “scale” (version 4.1.2) to normalize ranges between different cytokines and chemokines. The heatmap was created using the “heatmap.plus” package (version 1.3), with unsupervised hierarchical clustering of parameters performed using the “hclust” option. Correlation plots with Spearman rank correlation coefficient (r) between all paired parameters were created using the “corrplot” (version 0.92). Spearman rank two-tailed p-values were calculated using corr.mtest and graphed based on * p <0.05, **p <0.01, *** p <0.001, as described previously (14).

### Statistical analysis

Graphs and statistical analysis were performed at GraphPad Prism version 9.0.0 for Windows (GraphPad Software, CA, USA). Non-parametric statistical tests Mann-Whitney and Kruskall-Wallis with Dunn’s correction for multiple comparisons were applied to compare groups, and differences were considered statistically significant for p values ≤0,05. All authors assume responsibility for the accuracy and fidelity of the data and analyses.

## Results

### Study design and participants

To assess the cellular and humoral responses after immunization with CoronaVac, the initial consecutive 120 volunteers enrolled in a randomized, double-blind, placebo-controlled phase 3 clinical trial (NCT04456595) were selected based on vaccination status, age and specimen availability. The final cohort was primarily composed of 29 vaccinees and 4 placebos ranging from 18 to 59 years, and 24 vaccinees and 4 placebos over 60 years old (totalizing 53 vaccinees and 8 placebos). Seven vaccinated volunteers were excluded from further analysis due to lack of follow-up or positive COVID-19 diagnosis right after the first vaccine dose. Plasma and PBMC samples were obtained before vaccination (0d), 14 days after the first dose (14d, collected just before the second dose) and at other time points after the first vaccine dose (30d, 60d, 90d, 120d, 180d, 360d). Placebos were only evaluated before vaccination and there were no infections reported in this group during the follow-up.

In order to quantify the response magnitude achieved after vaccination, 17 SARS-CoV-2 infected, age-paired individuals were selected as positive controls (eight hospitalized patients due to moderate COVID-19 symptoms and nine outpatients with mild disease). Blood samples from the hospitalized patients are part of the SARS-CoV-2 neutralizing antibody investigation research project approved by local and national institutional review board (CAAE 34634620.1.1001.0068). The blood specimens from non-hospitalized patients were obtained from SARS-CoV-2 infected placebos enrolled in CoronaVac clinical trial and were collected after infection. Non-hospitalized patients were only included in the assessment of humoral response due to the lack of PBMC samples. The demographics of the included individuals are summarized in **Table 1**.

### CoronaVac induces CD4^+^ T cell responses sustained for at least 6 months

**Figure 1:**
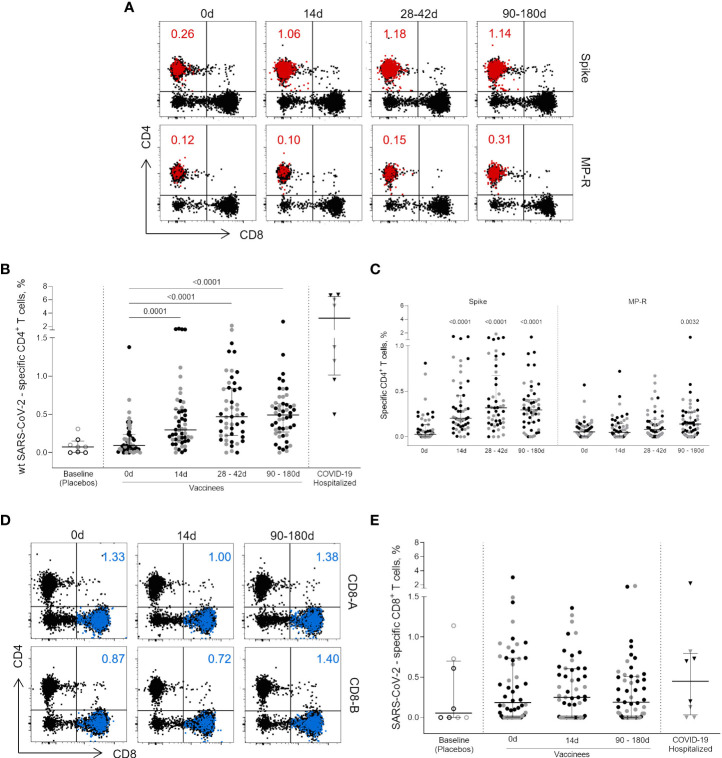
CD4^+^ and CD8^+^ circulating T cells activated after vaccination. **(A)** Representative flow cytometry plots showing the activated (OX40^+^CD25^+^CD137^+^) CD4^+^ T cells (red dots) after the stimulation with Wuhan/WH04/2020 (wt) SARS-CoV-2 Spike and non-spike (MP-R) peptides megapools (MPs). The total response is presented as the sum values obtained for Spike- and non-Spike-specific CD4^+^ designed MPs. **(B)** Frequency of activated SARS-CoV-2-specific CD4^+^ T cells before and after first and second vaccine doses. **(C)** Frequency of activated SARS-CoV-2-specific Spike and non-Spike (MP-R) CD4^+^ T cells before and after the first and second vaccine doses. **(D)** Representative FlowJo analysis of activated (CD69^+^CD137^+^) CD8^+^ T cells (in blue) at three time points after stimulation with SARS-CoV-2 peptides MPs targeting most prominent HLA class I alleles (CD8-A and CD8-B) **(E)** Frequency of activated SARS-CoV-2-specific CD8^+^ T cells before and after the first and second vaccine doses. The percentage of activated cells was determined by using a Boolean gate on the total live CD4^+^ or CD8^+^ T cells. In the scatter dot plot graphs: symbol colours represent age-groups (black: 18-59, gray: ≥60); symbol shapes represent different volunteer sub-groups (unfilled circle: placebos (n = 8), filled circle: vaccinees (n = 52), filled triangles: SARS-CoV-2 infected hospitalized individuals (n = 8), 19 days median of symptoms onset (IQR 17-22)). 0d: vaccinees baseline, day of first vaccine dose (n = 52). 14d: two-weeks after the first dose, day of the second vaccine dose (n = 52). 28-42d: from 28 42 days after the first vaccine dose (n = 49). 90-180d: from 90 to 180 days after the first vaccine dose (n = 51). Three individuals missed the 28-42d visit, one individual was excluded at the latest visit due to the detection of SARS-CoV-2 infection and one was excluded due to lack of baseline PBMC sample. Statistical comparisons using the Kruskall-Wallis with *post hoc* Dunn’s test against baseline values were used.

Peptides designed to overlap the Wuhan/WH04/2020-SARS-CoV-2 ORFeome (1) enabled the assessment of SARS-CoV-2-specific CD4^+^ and CD8^+^ T cell immunity by a cytokine-independent ex vivo T cell assay in cryopreserved PBMC samples obtained prior and after vaccination (**Figure 1**). The total SARS-CoV-2-specific CD4^+^ T cell response (OX40^+^CD25^+^CD137^+^, **Figure 1A**), presented as the sum values obtained for Spike and non-Spike CD4^+^ designed MPs, was detected 14 days after the first vaccine shot (p = 0.0001) (**Figure 1B**). In five individuals (four from the 18-59 years group and one from the ≥60 years group) SARS-CoV-2-specific CD4^+^ T cell response frequency reached more than 1% of total circulating CD4^+^ T cells with one vaccine dose, close to the frequency observed in some infected individuals (**Figure 1B**). Higher frequencies were detected between 28-42 days after first vaccine dose (14 and 30 days after the second dose; p <0.0001) and this magnitude of response was maintained from 90 to 180 days after first dose (75 to 165 days after second dose; p <0.0001) (**Figure 1B**). CD4^+^ T cells targeting the SARS-CoV-2 Spike ORF were the most representative cell subset of the total SARS-CoV-2-specific CD4^+^ T cell response at all time points evaluated after vaccination (p <0.0001) (**Figure 1C**). CD4^+^ T cell responses to the non-spike SARS-CoV-2 ORFeome (MP-R) were found significant only at later time points (90 to 180 days after first dose; p <0.0032) (**Figure 1C**). No significant SARS-CoV-2-specific CD8^+^ T cell responses were detected among vaccinees after the stimulation with SARS-CoV-2 MPs containing peptides targeting predicted CD8^+^ T cell epitopes of the most prominent HLA class I alleles (**Figures 1D, E**).

### Tfh and non-Tfh cell subsets are elicited after immunization with CoronaVac

**Figure 2:**
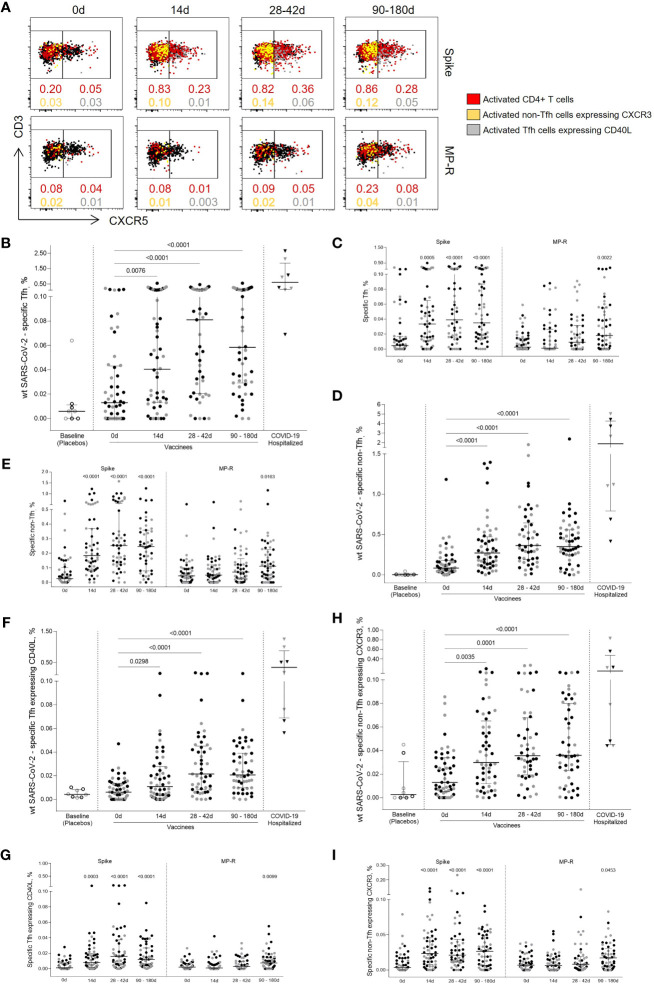
Specific SARS-CoV-2 Tfh and non-Tfh cells activation kinetics. **(A)** Representative flow cytometry plots showing Tfh (CD4^+^CD45RA^-^CXCR5^+^) and non-Tfh (CD4^+^CD45RA^-^CXCR5^-^) cells before and after the first and second vaccine doses. The red dots represent activated (CD25^+^CD137^+^OX40^+^) CD4^+^ T cells. The yellow dots represent activated (CD25^+^CD137^+^OX40^+^) non-Tfh cells expressing CXCR3; the gray dots represent activated (CD25^+^CD137^+^OX40^+^) Tfh cells expressing CD40L. **(B)** Frequency of activated SARS-CoV-2-specific Tfh cells. **(C)** Frequency of activated Tfh cells specific for SARS-CoV-2 Spike and MP-R peptides. **(D)** Frequency of activated SARS-CoV-2-specific non-Tfh. **(E)** Frequency of activated non-Tfh specific for SARS-CoV-2 Spike and MP-R peptides. **(F)** Frequency of activated SARS-CoV-2-specific Tfh expressing CD40L. **(G)** Frequency of activated Tfh expressing CD40L specific for SARS-CoV-2 Spike and MP-R peptides. **(H)** Frequency of activated SARS-CoV-2-specific non-Tfh expressing CXCR3. **(I)** Frequency of activated non-Tfh expressing CXCR3 specific for SARS-CoV-2 Spike and MP-R peptides. The percentage of cells co-expressing activation and other cell markers was determined by using a Boolean gate on the total live CD4+ T cells. In the scatter dot plot graphs: symbol colours represent age-groups (black: 18-59, gray: ≥60); symbol shapes represent different volunteer sub-groups (unfilled circle: placebos (n =8), filled circle: vaccinees (n = 52), filled triangles: SARS-CoV-2 infected hospitalized individuals (n = 8), 19 days median of symptoms onset (IQR 17-22)). 0d: vaccinees baseline, day of first vaccine dose (n = 52). 14d: two-weeks after the first dose, day of the second vaccine dose (n = 52). 28-42d: from 28 42 days after the first vaccine dose (n = 49). 90-180d: from 90 to 180 days after the first vaccine dose (n = 51). Three individuals missed the 28-42d visit, one individual was excluded at the latest visit due to the detection of SARS-CoV-2 infection and one was excluded due to lack of baseline PBMC sample. Statistical comparisons using the Kruskall-Wallis with *post hoc* Dunn’s test against baseline values were used.

To assess SARS-CoV-2-specific CD4^+^ T cell response polarization, circulating T follicular helper (Tfh) and non-Tfh cells frequencies were measured based on the expression of the CXCR5 chemokine receptor (**Figure 2A**). Both SARS-CoV-2-specific Tfh (CD45RA^-^CXCR5^+^) and non-Tfh (CD45RA^-^CXCR5^-^) cells frequencies had a significant increase after first vaccine dose (p = 0.0076 and p <0.0001, respectively), showing a greater increment after the 14-day boost (p <0.0001; **Figures 2B–E**). SARS-CoV-2-specific Tfh cell responses peaked 14 days after the second shot, subsequently dropping, although maintaining levels greater than those detected after the first vaccine dose (**Figure 2B**).

SARS-CoV-2-specific Tfh cells detected in post-vaccine time points were found to co-express CD40L, a T cell activation marker crucial to B cell maturation (**Figure 2F**). Similar to what was previously observed for total CD4^+^ T cell response, Spike-specific Tfh circulating cells expressing CD40L were the most predominant cell subset detected after the two-dose vaccination and were significantly increased in all post-vaccine timepoints compared to baseline (14 days, p <0.0003; 28-42 days and 90-180 days, p <0.0001, **Figure 2G**). Significant CD40L^+^ Tfh cell MP-R-specific responses were detected after 90 days of the first vaccine shot (p = 0.0099) (**Figure 2G**). The bulk response against SARS-CoV-2 peptides for both Tfh and non-Tfh cells were directed by the Spike peptides MPs (**Figures 2C, E**).

non-Tfh cells consist of heterogeneous cell populations that are often functionally stratified in humans by the expression of other chemokine receptors, including CXCR3 (8–10). CXCR3^+^ non-Tfh, Th1-polarized cells, were significantly increased after the first vaccine dose (p = 0.0035) reaching higher magnitude after second vaccine dose (p ≤0.0001) (**Figure 2H**). Over 70% of the vaccinees had the frequency of CXCR3^+^ non-Tfh cells specific for SARS-CoV-2 above median values detected at baseline and the majority of this response was driven by Spike-peptides (**Figure 2I**).

### Activated memory CD4^+^ cells are induced after the first vaccine dose and are sustained up to 6 months

**Figure 3:**
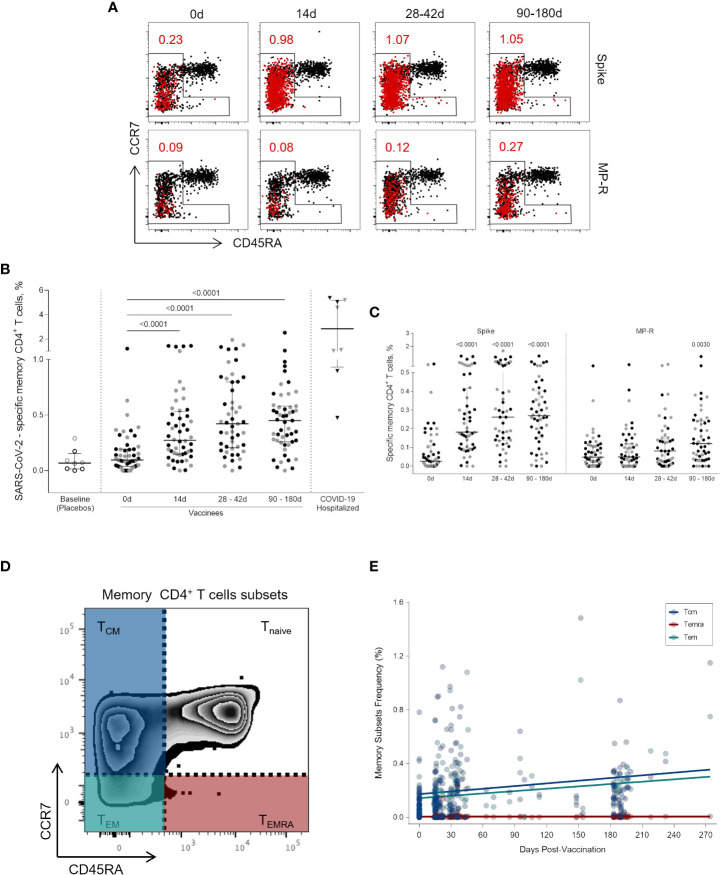
Activated memory CD4^+^ T cells specific for SARS-CoV-2 peptides. **(A)** Representative FlowJo analysis of activated (CD25^+^CD137^+^OX40^+^) memory T CD4^+^ T cells (in red) at four time points. Memory CD4^+^ T cells here encompass all cell subsets based on surface expression of CD45RA and CCR7 except the naive subset (CD45RA^+^CCR7^+^) **(B)** Frequency of activated memory CD4^+^ T cells specific for SARS-CoV-2 before and after the first and second vaccine doses. **(C)** Frequency of activated memory CD4^+^ T cells specific for SARS-CoV-2 Spike and MP-R peptides. **(D)** FlowJo analysis strategy example showing the subsets of memory CD4^+^ T cells: central memory (TCM) (CCR7^+^CD45RA^-^) in blue; effector memory (TEM) (CCR7^-^CD45RA^-^) in green; and terminally differentiated effector memory cells (TEMRA) (CCR7^-^CD45RA^+^) in red. **(E)** Distribution of the activated memory CD4^+^ T cell subsets among total SARS-CoV-2-specific CD4^+^ T cells. The percentage of cells co-expressing activation and other memory cell markers was determined by using a Boolean gate on the total live CD4^+^ T cells. In the scatter dot plot graphs: symbol colors represent age-groups (black: 18-59, grey: ≥60) and symbol shapes represent different volunteer sub-groups (unfilled circle: placebos (n = 8), filled circle: vaccinees (n = 52), filled triangles: SARS-CoV-2 infected hospitalized individuals (n = 8), 19 days median of symptoms onset (IQR 17-22)). 0d: vaccinees baseline, day of first vaccine dose (n = 52). 14d: two-weeks after the first dose, day of the second vaccine dose (n = 52). 28-42d: from 28 42 days after the first vaccine dose (n = 49). 90-180d: from 90 to 180 days after the first vaccine dose (n = 51). Three individuals missed the 28-42d visit, one individual was excluded at the latest visit due to the detection of SARS-CoV-2 infection and one was excluded due to lack of baseline PBMC sample. Statistical comparisons using the Kruskall-Wallis with *post hoc* Dunn’s test against baseline values were used.

Circulating SARS-CoV-2-specific memory CD4^+^ T cells (i.e., non-naïve CD4^+^CD45RA^+^CCR7^+^ T cells) (**Figure 3A**) were identified at a quite high rate early on after one vaccine dose (p <0.0001), and this frequency was increased and maintained over a six-months period (p <0.0001) (**Figure 3B**). The expansion of circulating SARS-CoV-2 memory CD4^+^ T cells (above the baseline median value) at 14 days after the first vaccine dose was found in 83% (43/52) of vaccinees. Between 28 and 42 days after the first dose the percentage of vaccinees with SARS-CoV-2 memory CD4^+^ T cells above the median basal level increased to 92% (45/49) and was maintained at this level in 88% of the individuals between 90 and 180 days (45/51). Memory T CD4^+^ cells activation was mainly triggered by Spike-derived peptide stimulation. The frequency of MP-R specific memory CD4^+^ cells significantly increased only at the latest time point evaluated (90 to 180 days after first shot, p = 0.0030) (**Figure 3C**).

To further characterize the SARS-CoV-2-specific memory CD4^+^ T cells detected after vaccination, we assessed the diversity of memory cell subsets (**Figure 3D**). Central memory (TCM) and effector memory (TEM) cells are the most representative memory cell subsets due to progressive frequency increase after first and second vaccine doses while no change was observed in terminally differentiated effector memory cells (TEMRA) (**Figure 3E**).

### CoronaVac shifts cytokine and chemokine pattern throughout time

Next, 35 cytokines and chemokines released in cell culture supernatant in response to Spike and non-Spike CD4^+^ T cells designed MPs were measured to characterize its profiles at early and later post-vaccination time points, stratified by the vaccine recipient’s age. To analyze the relation between age, time after vaccination and immune response, a pairwise Spearman rank correlation test was performed between all parameters, with unsupervised clustering (**Figure S1**.) and separated by time post-vaccination and age (**Figure S2**). The cytokine pattern changed throughout time; a greater range of cytokines and chemokines were cross-correlated by their upturn in expression after the second dose, which was maintained until the last time point (90 to 180 days) (**Figure S2**). Older vaccinees took longer to form a positive correlated cytokine and chemokine response, while adults had an escalation response after only one vaccine dose. Elderly individuals needed at least two doses, even though, by day 180, the response profiles were similar between both age stratified groups.

**Figure 4:**
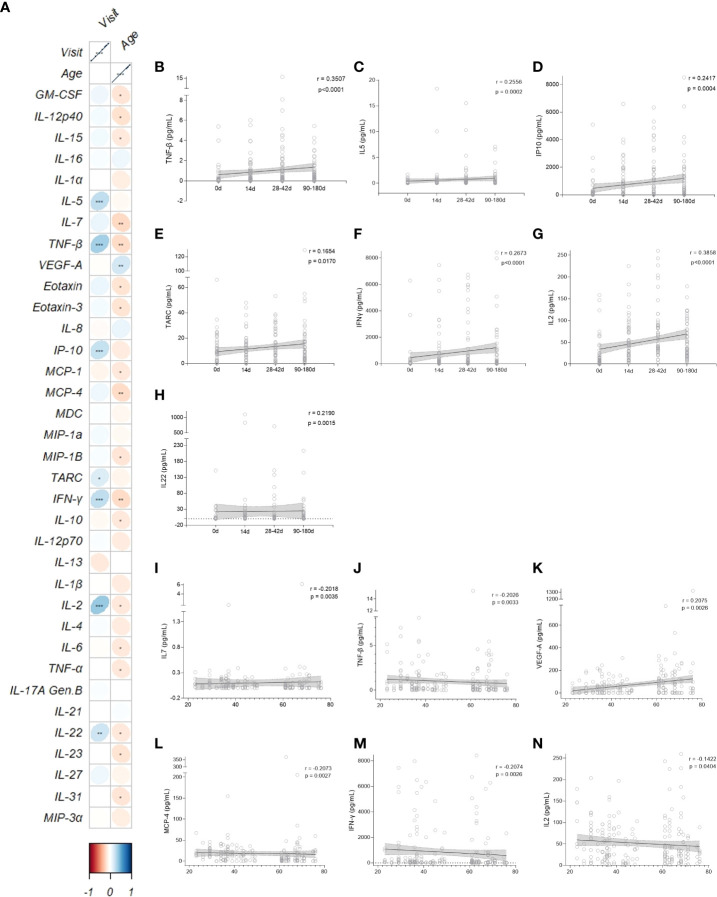
CD4+ T related Cytokine and Chemokine patterns after vaccination. **(A)** Correlation matrix between cytokines/chemokines and vaccine clinical trial visit and age. The upward slope of the ellipses shows positive correlations in blue whereas downward ones show negative correlations in red. Color intensities and sizes of ellipses are proportional to the absolute value of the corresponding Spearman correlation coefficients (legend at the bottom); figure generated with the R package corrplot, *p < 0.05, **p < 0.01, ***p < 0.001. Spearman’s correlation (95% confidence interval, in gray) between the most statistical relevant cytokines and the vaccine clinical trial visit (**B-H**, black and horizontal lines) and age of participants (**I-N**, black and vertical lines). 0d: vaccinees baseline, day of first vaccine dose (n = 52). 14d: two-weeks after the first dose, day of the second vaccine dose (n = 52). 28-42d: from 28 to 42 days after the first vaccine dose (n = 49). 90-180d: from 90 to 180 days after the first vaccine dose (n = 51). Three individuals missed the 28-42d visit, one individual was excluded at the latest visit due to the detection of SARS-CoV-2 infection and one was excluded due to lack of baseline PBMC sample. Statistical comparisons using the Kruskall-Wallis with *post hoc* Dunn’s test against baseline values were used. See also **Figures S1-S3**.

Data from the cytokine and chemokine measurements were summarized in a correlation plot crossing age and visit (**Figure 4**). Interestingly, 7 out of 35 cytokines (TNF-β, IL-5, IP-10, TARC, IFN-γ, IL-2 and IL-22) were positively correlated with time lapsed post-vaccination (Fig 4B-H). From those, all except IL-22 and TARC were in the same cluster, and IL-2 and TNF-β had the strongest correlations coefficients (r = 0.3815 and r = 0.3500, respectively) (**Figures 4B, G**). IL-22 and TARC were further away from the other cytokines because they had a lower statistically significant p-value (r = 0.2148, p = 0.0020 and r = 0.1549, p = 0.0270, respectively) (**Figures 4E, H**). Four out of the 35 cytokines (IL-7, TNF-β, MCP-4 and IFN-γ) were negatively correlated with age (**Figures 4I–N**); VEGF-A was the only one with a significant positive correlation with age (r = 0.2005, p = 0.0040) (**Figure 4K**). We also plotted the IL-2 correlation due to its interestingly strongest positive correlation with time point, which is seen to be the opposite when correlated with age (r = -0.1625, p = 0.0202) (**Figure 4N**).

Although cytokines and chemokines secreted in supernatants from activated cells in response to Spike and non-Spike MPs stimulation cannot be directly associated with specific cell populations, they may indicate functionality and polarization of the SARS-CoV-2-specific cell response (**Figure S3**). Adult vaccinees showed a consistent presence of IL-2, IL-1β, IFN-γ and TNF, indicative of a Th1 profile, as well as a more established expression of Th2 cytokines (IL-4 and IL-10). Modest Th17-polarized response was also detected, with a distinctive IL-17 and IL-27 positively correlation with specific T cell subtypes, whereas IL-22 is consistently and positively correlated with all T cell populations analyzed.

When stratifying by age group and throughout time there is a shift between cytokine expression and T cell population. The elderly vaccinees presented the same patterns but with modest correlation. Interestingly, VEGF-A, an angiogenic factor, changed from a negative correlation to a non-correlation status after the first dose in adults, yet had a strong negative correlation at the same time point in elderly vaccinees. Older individuals also achieved a positive correlation between IL-21 and Tfh cells after 180 days from the first dose, shifting from a strong negative correlation at baseline. Additionally, after 6 months of the two CoronaVac doses, adults continue to have SARS-CoV-2 specific memory and activated effector T cells, whilst elderly vaccinees have a diminished expression of Tfh CD40L^+^ cells, agreeing to what is seen in the literature (15). There is an interesting shift between cytokine expression and T cell population throughout time and stratified by age group (**Figure S3**).

### CoronaVac induces high levels of specific IgG against SARS-CoV-2 and VOCs, maintained up to one year

**Figure 5:**
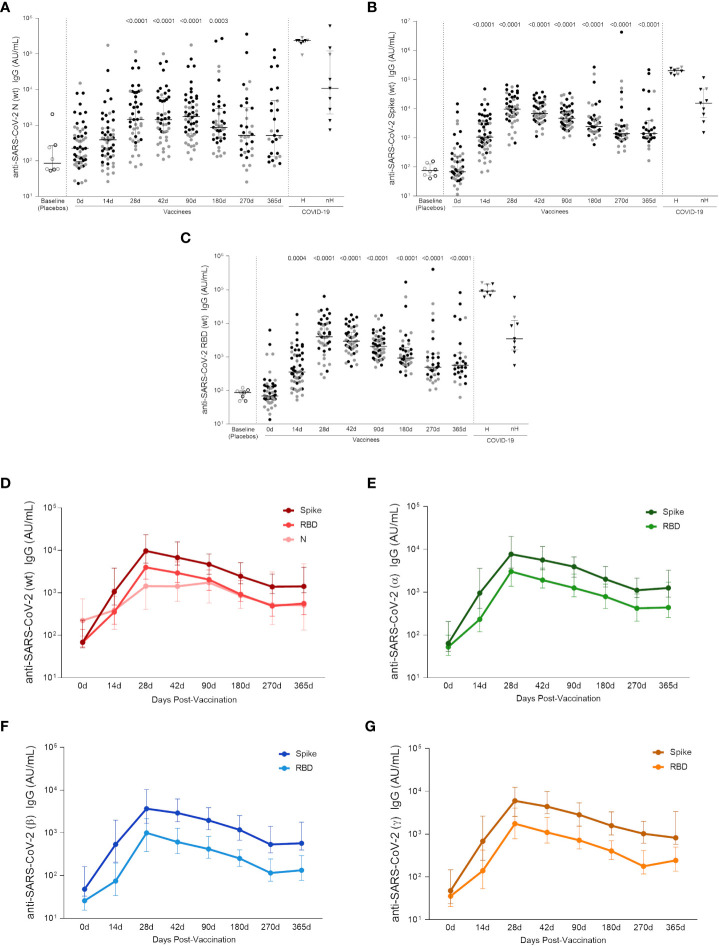
Specific wt-SARS-CoV-2 and VOCs strains IgG titers. **(A)** IgG levels measured against Nucleocapsid, **(B)** Spike and **(C)** RBD proteins from the Wuhan/WH04/2020-SARS-CoV-2 (wt). **(D)** IgG titers against S, RBD and N proteins from the Wuhan/WH04/2020-SARS-CoV-2 (wt). IgG levels against Spike and RBD proteins from **(E)** Alpha (B.1.117), **(F)** Beta (B.1.351) and **(G)** Gamma (P.1) strains. In the scatter dot plot graphs: symbol colors represent age-groups (black: 18-59, gray: ≥ 60) and symbol shapes represent different volunteer sub-groups (unfilled circle: placebos (n = 8), filled circle: vaccinees (n = 53), filled triangles: SARS-CoV-2 infected hospitalized (n = 8) (19 days median of symptoms onset (IQR 17-22)) or non-hospitalized individuals (n = 9) (46 days median of symptoms onset (IQR 34-65)). In **(D–G)** values are expressed as the median and the 25-75% IQR. AU: arbitrary units. 0d: vaccinees baseline, day of first vaccine dose (n = 53). 14d: two-weeks after the first dose, day of the second vaccine dose (n = 53). 28d: four-weeks after the first vaccine dose (n = 46). 42d: six-weeks after the first vaccine dose (n = 47). 90d: three-months after the first vaccine dose (n = 52). 180d: six-months after the first vaccine dose (n = 41). 270d: nine-months after the first vaccine dose (n = 34). 365d: one-year after the first vaccine dose (n = 27). Seven individuals missed the 28d visit, six missed the 42d visit, ten missed the 180d visit, fourteen missed the 270d visit and twenty-one missed the 365d visit. One individual was excluded from the 90d visit, two from the 180d visit, five from the 270d visit and five from the 365d visit due to the detection of SARS-CoV-2 infection. Statistical comparisons using the Kruskall-Wallis with post hoc Dunn’s test against baseline values were used. See also **Figure S4**.

As long-lasting SARS-CoV-2-specific Tfh cells were detected in the AIM assay, the next goal was to assess the levels of circulating antibodies against SARS-CoV-2 proteins in adult and elderly vaccinees, up to one year after the first vaccine dose. Analysis of both groups taken together showed a significant increase in the anti-Wuhan/WH04/2020-SARS-CoV-2 (wt-SARS-CoV-2) IgG titers 14 days after the first vaccine shot for Spike and RBD proteins but not for the N antigen. Two weeks after the two-dose vaccination scheme (i.e., 28 days after first vaccine dose) IgG levels against wt-SARS-CoV-2 N, Spike and RBD proteins were significantly higher compared to baseline levels (p <0.0001, **Figures 5A–C**). Through one year follow-up SARS-CoV-2 N-specific IgG titers were relatively stable from 28 to 90 days after first vaccine shot, going gradually down in the next 6 months until one year after vaccination (**Figure 5A**). Wuhan/WH04/2020-SARS-CoV-2 S- and RBD-specific IgG titers peaked from 28 to 42 days at similar levels found in non-hospitalized infected individuals (46 days median after symptoms onset, IQR 34-65) (**Figures 5B, C**). Between 90 and 270 days S- and RBD-specific IgG levels showed a slight decay, reaching stability point from 270 to 365 days after first vaccine dose at significantly higher levels compared to baseline (p <0.0001) and the placebo group (p <0.0001) (**Figures 5B, C**). The same IgG response profile was found when analyzing antibodies against S and RBD proteins of Alpha, Beta and Gamma VOCs (**Figures 5E–G**). IgG response peaked at 28 days after first vaccine dose, and even though it subsequently presented a slight decrease, antibody levels remained significantly higher after one year of vaccination compared to baseline (median value >10^2^ AU/mL, and p <0,0001 for all strains, **Figure S4**). It should be noted that the method used to calculate anti-Spike, anti-RBD and anti-N IgG titers against Wuhan/WH04/2020 and VOCs is based on an unique standard curve for each variant peptide target, and they cannot be directly compared. Nevertheless, the data shows that all titers have a similar expansion kinetics profile (**Figures 5D–G**).

By assessing the Wuhan/WH04/2020-SARS-CoV-2 and VOCs IgG responses in each age group, a higher level of antibodies against S and RBD proteins were found among adult vaccinees when compared to the elderly individuals between 14 to 42 days after first vaccine shot. From 90 to 365 days after first dose, similar Wuhan/WH04/2020-SARS-CoV-2 and VOCs IgG levels were observed between both age groups (**Figure S4**). No significant increase in IgG response against Wuhan/WH04/2020-SARS-CoV-2 N protein was detected among elderly vaccinees, whereas in vaccinated adults significant N-specific IgG levels were found between 28 and 180 days after first dose.

## Discussion

COVID-19 vaccines that elicit protective immune responses are essential to prevent SARS-CoV-2 infection and mitigate the COVID-19 morbidity and mortality. The aim of this study was to evaluate the immune response to CoronaVac by measuring the T cell activation kinetics after re-stimulation with wt SARS-CoV-2 peptides, the cytokine and chemokine profile of the T cell culture supernatant and the IgG-specific levels against the Wuhan/WH04/2020 strain and VOCs antigens. The results add understanding on how this vaccine builds and maintains the immune response against SARS-CoV-2.

Multiple lines of evidence support the critical roles of T cells in mounting immune responses to COVID-19. It is well-known that T cells can engage several antigen epitopes providing a broader protection against the virus (16). SARS-CoV-2-specific peptides, which have been recognized by CD4^+^ and CD8^+^ T cells of exposed donors (1), allowed the detection of specific T cell responses even in individuals without detectable antibody responses, thereby providing evidence for T cell immunity upon vaccination. In line with previously published data showing stronger SARS-CoV-2-specific CD4^+^ T cell response compared to CD8^+^ response in individuals recovering from COVID-19 infection (17) and after mRNA vaccination (18), a significant CD4^+^ T cell response triggered by Spike and non-Spike SARS-CoV-2 peptides was detected in peripheral blood cells of a subset of adults and elderly subjects vaccinated with CoronaVac. The data shown here indicates that CD4^+^ T cell may target different SARS-CoV-2 epitopes and suggest that the presence of additional SARS-CoV-2 antigens, such as M and N, in future vaccine formulations would enable it to better mimic the SARS-CoV-2-specific CD4^+^ T cell response seen in naturally infected patients.

Although a modest enhancement of activated (CD69^+^ CD137^+^) CD8^+^ T cells was seen following vaccination, no significant SARS-CoV-2-specific CD8^+^ T cell responses were detected in our study, corroborating with recent findings of Coronavac vaccinees in Chile by Bueno et al. (9). It does not directly support other studies assessing cellular response induced by other COVID-19 vaccines (13,19–21), however the frequencies of circulating SARS-CoV-2-specific CD8^+^ T cells were still found to be relatively low (20,21). Of note, SARS-CoV-2-specific CD8^+^ T cell responses are undetectable in approximately 30% of convalescent COVID-19 cases, even when testing the full SARS-CoV-2 ORFeome of epitopes (22). Nevertheless, it is not possible to assert that those cases have no important CD8^+^ T cell response against SARS-CoV-2 infection, since they might have CD8^+^ T cell responses triggered by untested epitopes. Alternatively, it has been also speculated that SARS-CoV-2-specific CD8^+^ T cell responses are undetectable in the peripheral blood as they are mostly antigen-specific tissue-resident memory T cells, residing in the lung or upper respiratory tract tissues (23–25).

A panel of relevant cytokines and chemokines was also measured in cell supernatant after SARS-CoV-2 peptide stimulation to better characterize the immune response induced by the vaccine. Although it is not possible to ensure that the molecules were produced by specific T cells, they may be indicative of the functionality and polarization of the vaccine-induced response. Besides IFN-γ and IL-2, various other cytokines were also positively correlated with time post-vaccination and specific effector T cell populations, leading us to assume that the two-dose CoronaVac regimen is able to stimulate a balanced response between Th1 (IFN-γ, IL-2, TNF, IP-10), Th2 (IL-5, IL-10, TARC) and Th17/Th22 (IL-22) responses, complementing the activated specific cellular response of Tfh, non-Tfh and memory cells, which is, likewise, positively correlated with time post-vaccination, as also seen after SARS-CoV-2 infection (26,27). Particularly, IFN-γ and IL-2 production, mostly known to characterize a Th1 profile, were associated with a better outcome for SARS-CoV-2 infected patients (28) and their increase was seen after the two-dose regimen of several approved COVID-19 vaccines (19). Although IP-10 has been associated with impaired cell response during SARS-CoV-2 infection, this association usually comes with other proinflammatory cytokines, such as IL-6 and IL-8 (14), which had no significant correlation with time post-vaccination in our cohort.

Considering the age difference between groups, a less intense immune response was seen amid the two groups, and known inflammatory markers such as IL-6, TNF-α/β, MIP-β and IFN-γ were negatively correlated with increasing age, possibly related to immune senescence, but not with a chronic exacerbated inflammatory state often seen in elderly individuals (29). In fact, older individuals mounted a similar response after receiving the two CoronaVac doses, only with a few weeks of delay when compared with younger adults. The only cytokine positively correlated with age was the angiogenesis factor VEGF-A, but its age-related increase is not always related to a non-healthy state (30,31), as some studies hypothesize that it could be also beneficial to the process of aging (31).

Regarding the cellular response, a significant percentage of the circulating SARS-CoV-2-specific CD4^+^ T cells detected after two doses of CoronaVac exhibited a Tfh phenotype, similar to those observed following mRNA vaccination (18) and infection (32). Tfh cells are CD4+ lymphocytes specialized in regulating the adaptive immune response in germinal centers by enabling the selection of specific high affinity B cells and modulating affinity maturation in infection and vaccination (33). Therefore, Tfh cells are crucial for establishing durable humoral immunity, by helping structuring antibodies generation. Mudd and colleagues (34) demonstrated a significant correlation between the size of the germinal center B cell population in lymph nodes and the total Tfh cell population frequency following mRNA vaccination. Using a Spike immunodominant epitope, the authors showed an increase of specific Tfh in the blood of vaccinated individuals, peaking 28 days after the first dose. Interestingly, in our study we observed that not only circulating Tfh cells, but also the antibodies titers against Wuhan/WH04/2020-SARS-CoV-2 Spike and RBD antigens peaked between 28 and 42 days after CoronaVac first dose. Despite this, significant levels of IL-21, a cytokine involved in Tfh differentiation (35) were not detected in cell supernatant at these timepoints. One explanation for this may be that the cell supernatant was collected 24h after stimulation, and there is the possibility that the peak concentration of IL-21 was missed. Nevertheless, a positive correlation between IL-21 concentration and Tfh cells was seen in the elderly group, going from a negative correlation at baseline to a positive correlation with SARS-CoV-2-specific Tfh cells (including the Tfh cells expressing CD40L) 180 days post-vaccination. As this cytokine is known to differentiate Tfh cells (36), this could be an indication that this population is being recruited to stimulate B cells and generate antibody response.

As circulating antibodies levels against Wuhan/WH04/2020 and VOCs strains reached its highest on day 28 after first dose, we showed that bAbs against Wuhan/WH04/2020 Spike and RBD proteins had at least one year durability, a profile also seen post-infection, but not with such longevity (37), and had also a similar median range as identified in our non-hospitalized cohort with 46 days post-symptoms onset. Although antibodies against the N protein showed a relevant decrease after 6 months, not seen in infected individuals (37), about half of the individuals remained above the median levels until 1 year after first vaccine dose. To our knowledge this is the first study to provide such a long-lasting bAbs response for vaccinated individuals. A similar kinetics was observed for the bAbs against all Spike and RBD VOCs (Alpha, Beta and Gamma) proteins levels when its decreasing stopped at 9 months and remained stable still above the basal IgG level throughout 1 year after first dose, different from the diminishing tendency seen in another study for the IgG levels against Beta, Gamma and Delta Spike targets in non-hospitalized individuals after 4 months of COVID-19 diagnose (38).

Stratifying the age groups, we noticed that elderly vaccinees delayed to reach significant bAbs levels for wt and VOCs, kept at lower levels compared to those seen in younger adults and dropped at earlier time points, possibly being associated with the diminished frequency and response of naive circulating B and T cells in elderly individuals (39), and in accordance with previous studies (6,40).

In addition to help B cell and antibody responses, eliciting broad and long-lasting antiviral immunity requires the enrollment of CD4^+^ T cells and the generation of effective T cell memory (41) essential for protection against future infections. SARS-CoV-2 memory CD4^+^ T cells were detected in almost all subjects after receiving the two-dose regimen of CoronaVac, as well as seen in a cohort of mostly non-hospitalized patients with COVID-19 (37). In contrast to the memory subset proportion (TCM followed by TEM subset along the 6 months) seen here after CoronaVac vaccination and also after mRNA vaccination (18), COVID-19 infected individuals showed an inverse proportion, being TEM the most frequent for 6 months, out passed by the TCM subset after this timeframe (37). These data suggest that vaccination with CoronaVac elicits an early sustained memory immunity compared to infection, since TEM cells are associated with a more immediate defense.

This study has some limitations including the relatively small sample size that was restrained by IRB and resource restrictions for additional blood volume drawn at the time of the study concept. Despite the small number of participants, we were able to consistently assess the immune response in post-vaccination timepoints with very few samples missing in later follow-up visits due to COVID-19 diagnosis or limited cell availability. This work was produced with a cohort that was not exposed to Delta and Omicron variants at the time of vaccination and the period following it (2020 – 2021), so it was not possible to evaluate the CoronaVac immune response against these VOCs, which now have outnumbered most cases worldwide. These limitations should be addressed in future studies, with large cohorts.

In summary, the results shown herein provide evidence that CoronaVac is capable to induce a long-term antigen-specific CD4^+^ T cell response, to induce an effector cytokines pattern with reduced inflammation in older people and lead to high-titer antibody responses against Wuhan/WH04/2020 SARS-CoV-2 and VOCs strains in adults and elderly individuals and also highlight the importance of the two-dose regimen to establish a robust cellular and humoral response.

## Data availability statement

The original contributions presented in the study are included in the article/**Supplementary Material**. Further inquiries can be directed to the corresponding author.

## Ethics statement

The studies involving human participants were reviewed and approved by Brazilian National Research Ethics Council (CONEP), CAAE 34634620.1.1001.0068. The patients/participants provided their written informed consent to participate in this study.

## Author contributions

PC, EK and CS conceptualized the study. PC, AG, DW, AS and CS developed the applied methodologies; CC, MM, JD, MT, ACS, AO, AFG and CS performed the experiments and analyzed data. LF, AF and EP contributed with volunteers recruiting, sample collection and demographic data gathering. AG, DW and AS developed the peptides used in the study. PC, CC, MM, JD, MT and CS wrote the original Draft. AG, DW, AS, RS, EK and CS reviewed and edited the manuscript. EK and CS supervised the study. All authors contributed to the article and approved the submitted version.

## Funding

This work has been supported by Fundação Butantan,Instituto Butantan and São Paulo Research Foundation (FAPESP) (grants 2020/10127-1 and 2020/06409-1). This work was additionally supported by NIH contract 75N93019C00065 (AS, DW). PATH facilitated reagent donations for this work with support by the Bill & Melinda Gates Foundation (INV-021239). CS was supported by a scholarship from HCFMUSP with funds donated by NUBANK under the #HCCOMVIDA scheme.

## Acknowledgments

We would like to express our thanks to the study subjects for their participation and desires to forward our scientific work. We would also like to acknowledge all individuals from the PROFISCOV study group, especially Wellington Briques (MD, MBA) and Gecilmara Salviato Pileggi (MD, MSc, PhD), for their scientific and technical assistance. Under the grant conditions of the foundation, a Creative Commons Attribution 4.0 generic License has already been assigned to the Author Accepted Manuscript version that might arise from this submission.

## Conflict of interest

Employees of Fundação Butantan and Instituto Butantan participated in the clinical trial design and data collection. Author AS is a consultant for Gritstone Bio, Flow Pharma, ImmunoScape, Moderna, AstraZeneca, Avalia, Fortress, Repertoire, Gilead, Gerson Lehrman Group, RiverVest, MedaCorp, and Guggenheim. The La Jolla Institute (LJI) has filed for patent protection for various aspects of T cell epitope and vaccine design work.

The remaining authors declare that the research was conducted in the absence of any commercial or financial relationships that could be construed as a potential conflict of interest.

## Publisher’s note

All claims expressed in this article are solely those of the authors and do not necessarily represent those of their affiliated organizations, or those of the publisher, the editors and the reviewers. Any product that may be evaluated in this article, or claim that may be made by its manufacturer, is not guaranteed or endorsed by the publisher.
