# Supplementary material for: Humoral and cellular immune responses to CoronaVac up to one year after vaccination
Source: Front Immunol. 2022 Oct 21;13:1032411. doi: 10.3389/fimmu.2022.1032411 (PMC9634255; doi:10.3389/fimmu.2022.1032411)
Supplement: Supplementary file 1 [file Image_1.pdf]

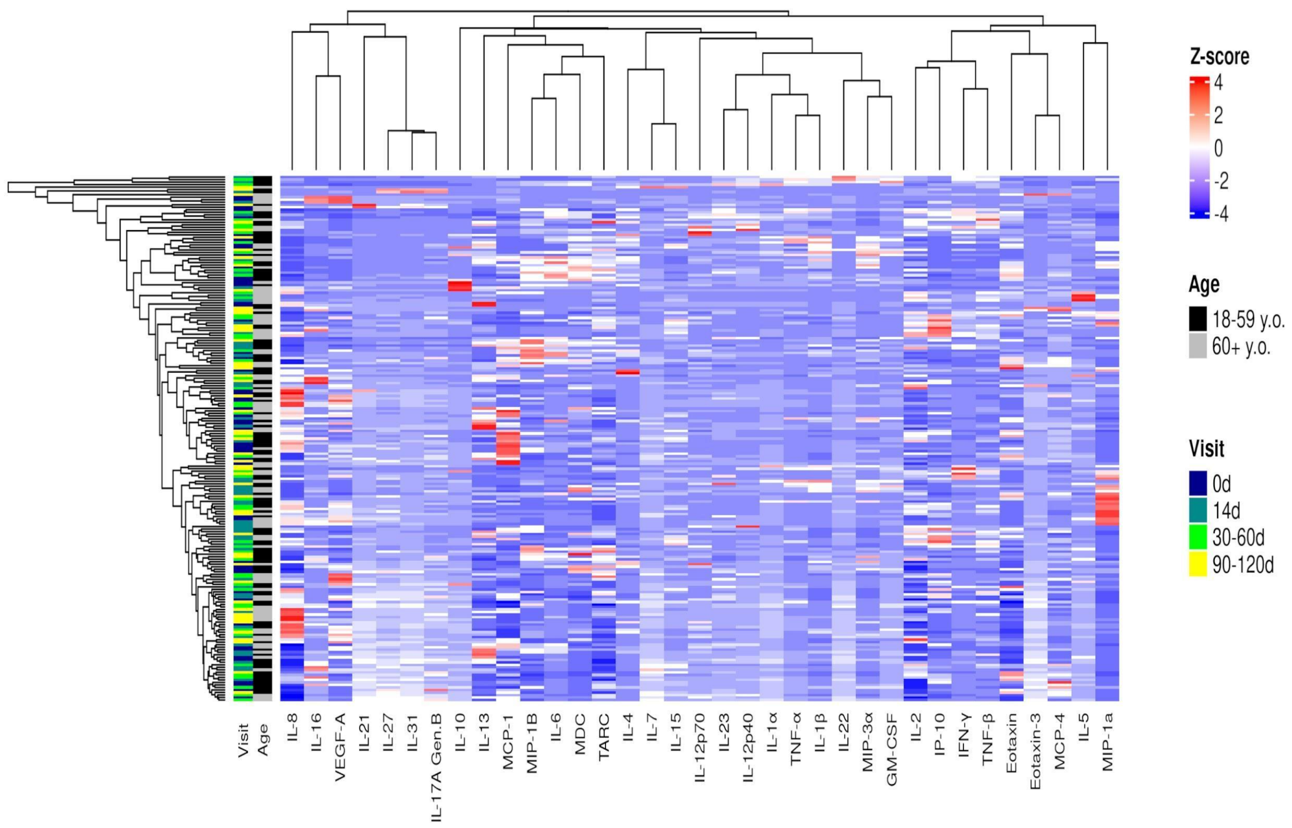

Supplementary Figure 1. Cytokine and chemokine clustering in CoronaVac vaccines. Heatmap represents an unsupervised clustering of the electrochemiluminescence assay of 35 cytokines and chemokines (columns) in 53 vaccine recipients (rows). Volunteers are separated by two age groups (black: from 18 to 59 years of age; grey: > 60 years) and four time points (from blue to yellow, respectively: 0d (before vaccine), 14d after the first dose, 30-60d after first dose and 90-120 after first dose). Data was standardized through the z-score and unsupervised clusterings were calculated using the “hclust” option of the “heatmap.plus” R package and data was visualised through the same package.
