# Supplementary material for: Humoral and cellular immune responses to CoronaVac up to one year after vaccination
Source: Front Immunol. 2022 Oct 21;13:1032411. doi: 10.3389/fimmu.2022.1032411 (PMC9634255; doi:10.3389/fimmu.2022.1032411)
Supplement: Supplementary file 2 [file Image_2.pdf]

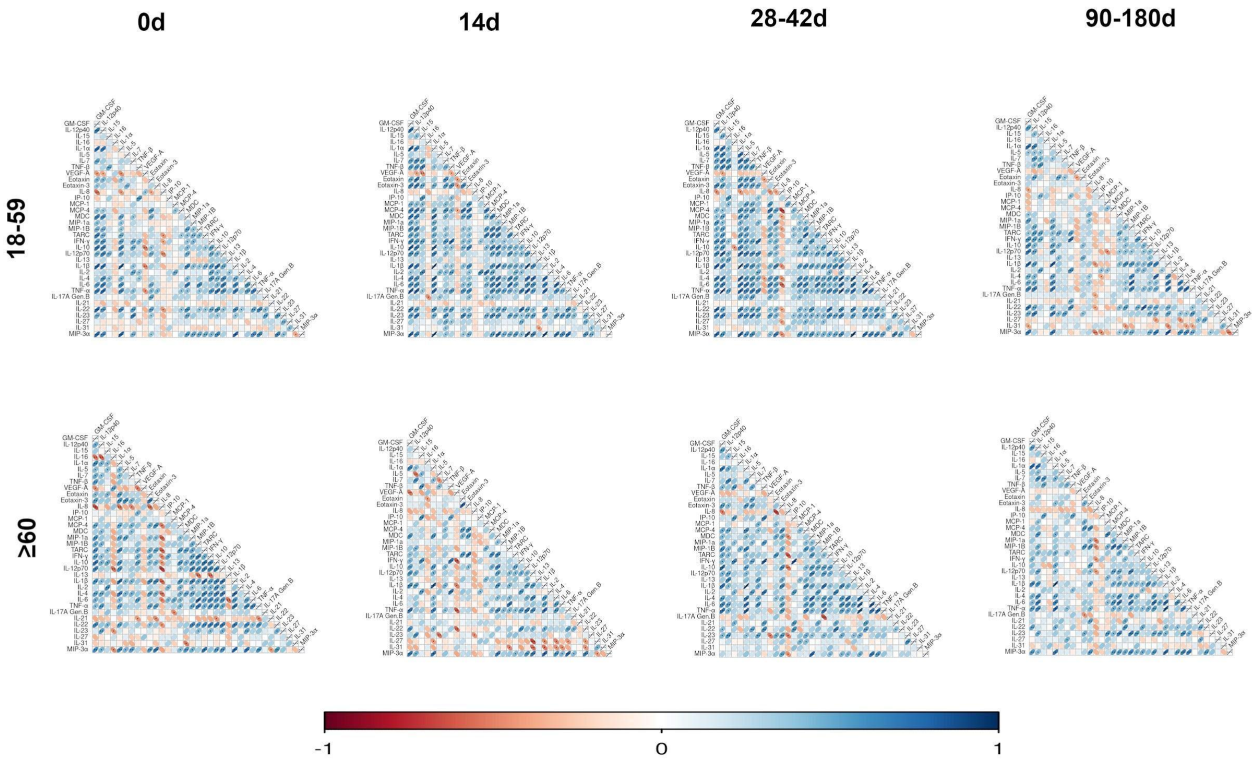

Supplementary Figure 2. Related to Figure 4. Cytokine and chemokine pattern after vaccination in adults and elderly vaccinees. Correlation plots of cytokines and chemokines produced upon vaccination, at different time points. The upward slope of the ellipses shows positive correlations in blue whereas downward ones show negative correlations in red. Colour intensities and sizes of ellipses are proportional to the absolute value of the corresponding Spearman rank order correlation coefficients are shown from red (-1.0) to blue (1.0), as indicated by the legend at the bottom. Graphs were generated with the R package 'corrplot'. *$p < 0.05$, **$p < 0.01$, ****$p < 0.0001$. 0d: vaccinee baseline, day of first vaccine dose. d: number of days after the first dose. 14d: day of the second dose.
