# Supplementary material for: Humoral and cellular immune responses to CoronaVac up to one year after vaccination
Source: Front Immunol. 2022 Oct 21;13:1032411. doi: 10.3389/fimmu.2022.1032411 (PMC9634255; doi:10.3389/fimmu.2022.1032411)
Supplement: Supplementary file 3 [file Image_3.pdf]

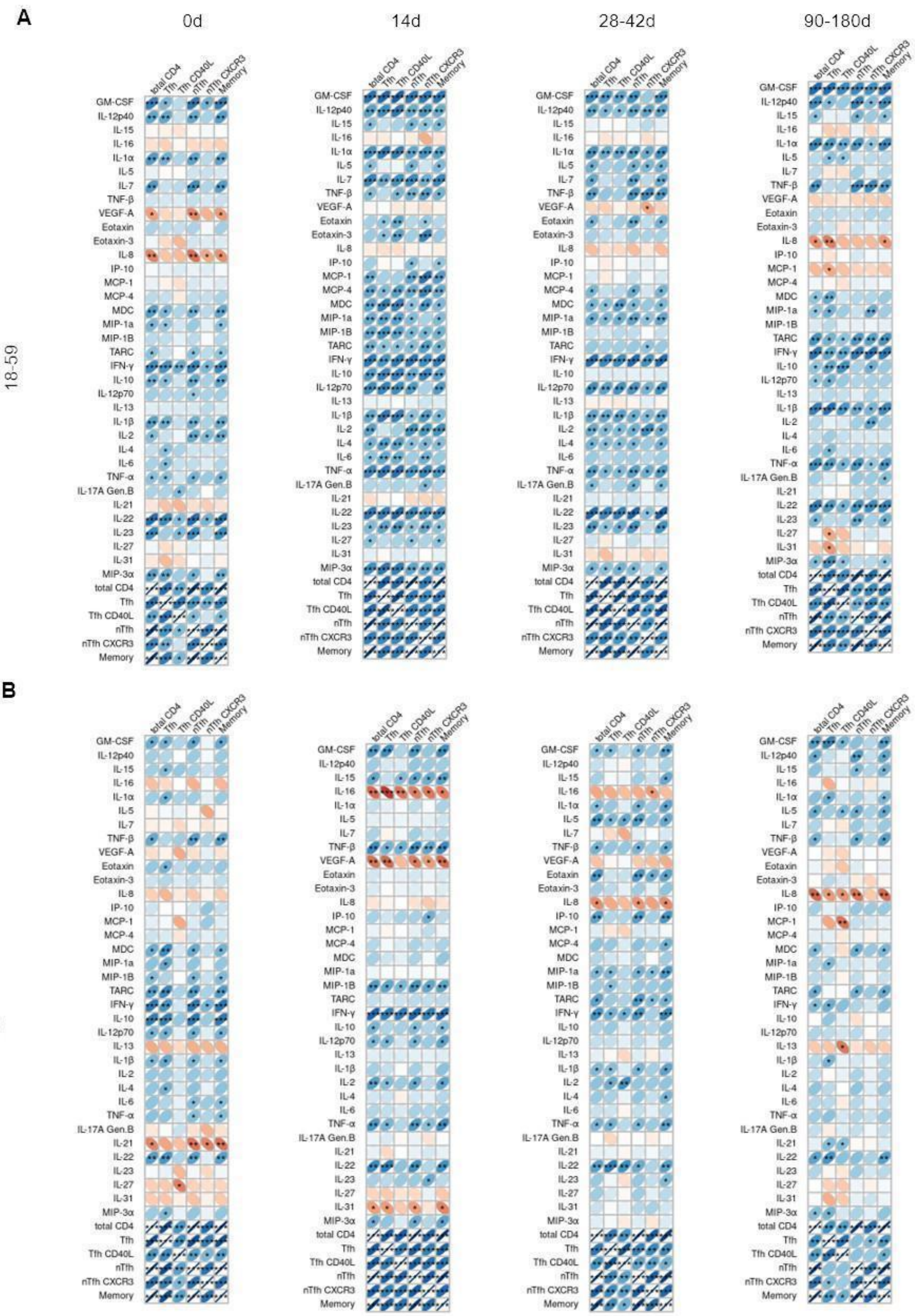

Supplementary Figure 3. Correlation between cytokine and chemokine concentrations and the expression of T cell populations. Correlation plots of cytokines and chemokines with different T cell populations. The correlation is shown between cytokines, chemokines and T cell population in 18-59 years old (A) and ≥60 years old (B) volunteers, according to the time

after vaccination, starting at the baseline (0d, day of first vaccine dose) until 180 days after the first dose. At 14d volunteers received the second vaccine dose. The upward slope of the ellipses shows positive correlations in blue whereas downward ones show negative correlations in red. Colour intensities and sizes of ellipses are proportional to the absolute value of the corresponding Spearman rank order correlation coefficients are shown from red (-1.0) to blue (1.0), as indicated by the legend at the bottom. Graphs were generated with the R package ‘corrplot’. *p < 0.05, **p < 0.01, ****p < 0.0001. d: number of days after the first dose.
