# Supplementary material for: Humoral and cellular immune responses to CoronaVac up to one year after vaccination
Source: Front Immunol. 2022 Oct 21;13:1032411. doi: 10.3389/fimmu.2022.1032411 (PMC9634255; doi:10.3389/fimmu.2022.1032411)
Supplement: Supplementary file 4 [file Image_4.pdf]

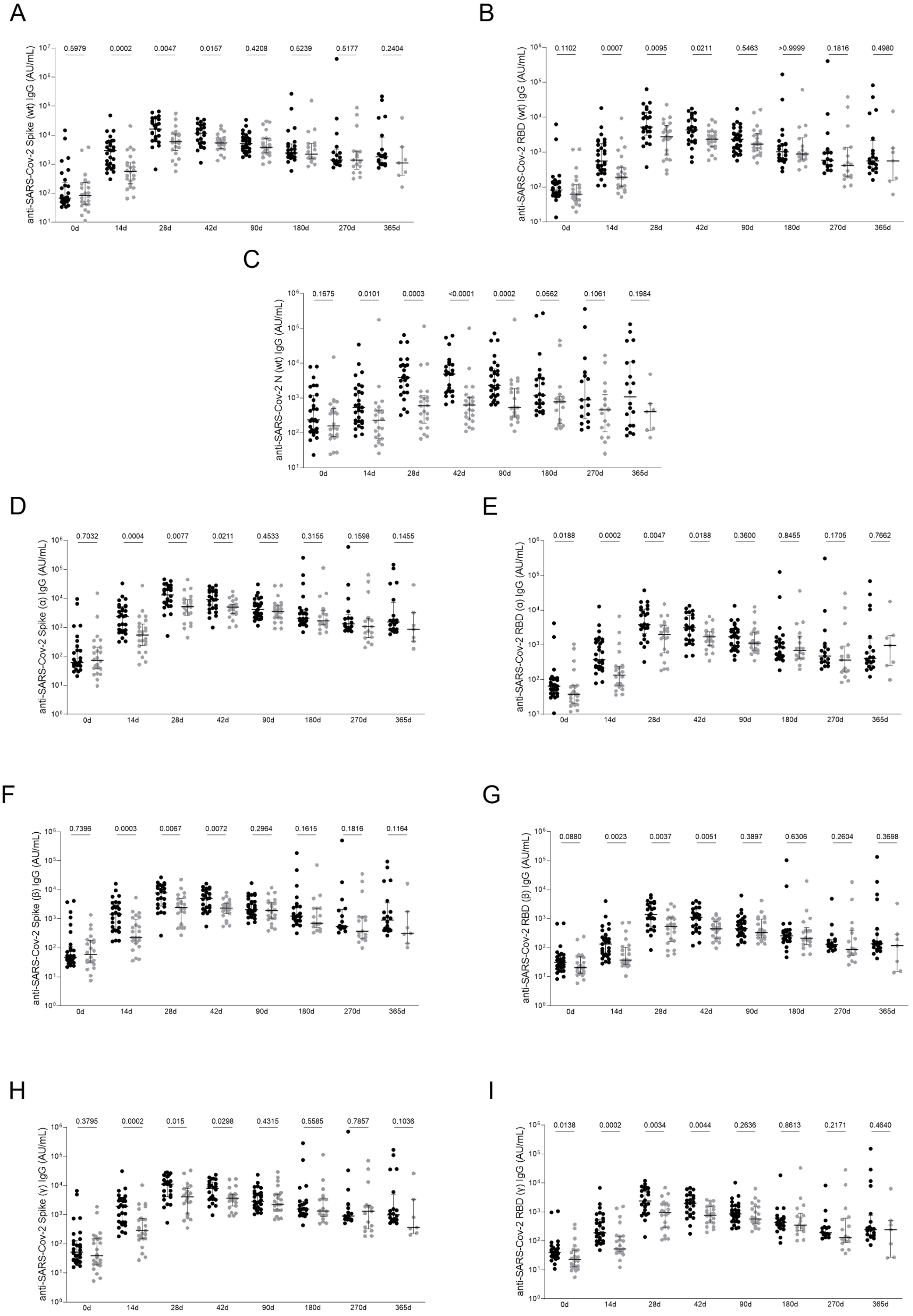

Supplementary Figure 4. Specific wt and VOCs SARS-CoV-2 strains IgG titers through one-year follow-up. Graphs show anti-SARS-CoV-2 IgG levels in AU/mL from vaccinees

throughout one year. Symbol colours represent age-groups (black: 18-59, grey: ≥60). 0d: vaccinee baseline, day of first vaccine dose (n = 29 adults/24 elderly). 14d: two-weeks after the first dose, day of the second vaccine dose (n = 29 adults/24 elderly). 28d-365d: number of days after the first dose (28d: n = 24 adults/22 elderly; 42d: n = 24 adults/23 elderly; 90d: n = 29 adults/23 elderly; 180d: n = 23 adults/18 elderly; 270d: n = 17 adults/17 elderly; 365d: n = 20 adults/7 elderly). Mann-Whitney test was performed to compare both age groups in each visit, and p-values are noted above each pair. IgG levels were measured for (A) wild-type Spike protein; (B) wild-type RBD protein; (C) wild-type nucleocapsid protein; (D-E) Alpha Spike and RBD proteins; (F-G) Beta Spike and RBD proteins and (H-I) Gamma Spike and RBD proteins.
