# Supplementary material for: Humoral and cellular immune responses to CoronaVac up to one year after vaccination
Source: Front Immunol. 2022 Oct 21;13:1032411. doi: 10.3389/fimmu.2022.1032411 (PMC9634255; doi:10.3389/fimmu.2022.1032411)
Supplement: Supplementary file 5 [file Table_1.pdf]

Supplementary Table 1. Antibodies and fluorochromes used in the activation induced marker (AIM) assay.

Fluorochrome	AIM T $CD4^+$ cells	AIM T $CD8^+$ cells
BB515	CD3 (UCHT1)	CD3 (UCHT1)
APC-H7	CD4 (RPA-T4)	CD4 (RPA-T4)
PerCP CY5,5	CD8 (SK1)	CD8 (SK1)
APC	CD45RA (HI100)	CD45RA (HI100)
PE-CY7	CD25 (M-A251)	-
BV711	CXCR3 (1C6)	CXCR3(1C6)
BV421	CCR7 (G043H7)	CCR7 (G043H7)
Alexa Fluor 700	CD40/CD154 (24-31)	-
APC-R700	-	CD38 (HIT2)
BV510	CD137 (4B4-1)	CD137 (4B4-1)
BV605	CXCR5 (RF8B2)	-
PE	OX40 (Ber-ACT35)[a]	CD69 (FN50)
PE-CF594	Live-Dead[b]	Live-Dead[b]

[a]All antibodies were acquired from BD Biosciences, excepting from anti-OX40 (Biolegend). Clones were described in parenthesis. [b]Cell viability marker, Live/Dead, CF-594, Life Technologies
