# Supplementary material for: Humoral and cellular immune responses to CoronaVac up to one year after vaccination
Source: Front Immunol. 2022 Oct 21;13:1032411. doi: 10.3389/fimmu.2022.1032411 (PMC9634255; doi:10.3389/fimmu.2022.1032411)
Supplement: Supplementary file 6 [file Table_2.pdf]

Supplementary Table 2. Biomarkers detected in the electrochemiluminescence binding assays, according to the MSD® (Meso Scale Discovery, MD, USA).

Chemokine Panel		Cytokine Panel		Proinflammatory Panel		Th17 Panel	
**Biomarkers**	**Detection range (pg/mL)**	**Biomarkers**	**Detection range (pg/mL)**	**Biomarkers**	**Detection range (pg/mL)**	**Biomarkers**	**Detection range (pg/mL)**
Eotaxin	12.3 - 1120	GM-CSF	0.842 - 750	IFN-γ	1.76 - 938	IL-17A Gen. B	5.86 - 1950
MIP-1β	1.88 - 520	IL-1α	2.85 - 278	IL-1β	0.646 - 375	IL-21	6.12 - 650
Eotaxin-3	10.2 - 3700	IL-5	4.41 - 562	IL-2	0.890 - 938	IL-22	2.78 - 325
TARC	3.32 - 1120	IL-7	0.851 - 563	IL-4	0.218 - 158	IL-23	4.6 - 3250
IP-10	1.37 - 500	IL-12/IL-23p40	1.32 - 2250	IL-6	0.633 - 488	IL-27	38.7 - 13000
MIP-1α	13.8 - 743	IL-15	0.774 - 525	IL-8[a]	0.591 - 375	IL-31	4.22 - 650

IL-8 (HA)	713 - 43400	IL-16	19.1 - 1870	IL-10	0.298 - 233	MIP-3α	0.750 - 325
MCP-1	1.09 - 375	IL-17A*	3.19 - 3650	IL-12p70	1.22 - 315	-	-
MDC	88.3 - 3700	TNF-β	0.465 - 458	IL-13	4.21 - 353	-	-
MCP-4	10 - 469	VEGF-A	7.70 - 562	TNF-α	0.690 - 248	-	-

[a]IL-8 from the Proinflammatory Panel and IL-17A from Cytokine Panel were excluded from the analysis due to being repeated in the Chemokine Panel and Th17 Panel, respectively. According to the manufacturer, IL-8 (HA) from the Chemokine Panel is validated and its use is recommended when high levels are anticipated and IL-17A Gen B is optimized for a more sensitive detection in multiple sample types, such as serum, plasma, urine, cell culture supernatants, and PBMCs.
