# Supplementary material for: Humoral and cellular immune responses to CoronaVac up to one year after vaccination
Source: Front Immunol. 2022 Oct 21;13:1032411. doi: 10.3389/fimmu.2022.1032411 (PMC9634255; doi:10.3389/fimmu.2022.1032411)
Supplement: Supplementary file 7 [file Presentation_1.pdf]

São Paulo PROFISCOV Study Group

Instituto Butantan, São Paulo, SP, Brazil

Clinical Trials and Pharmacovigilance Centre

Ricardo Palacios, Mônica Tilli Reis Pessoa Conde, Roberta de Oliveira Piorelli, Elizabeth González Patiño, Hugo Alberto Brango García, Joane do Prado Santos, Rodrigo Piske Finotto, Ana Paula Batista, Camila Santos Nascimento Albuquerque, Flávia Marilia Cestari Magalhães, Carolina de Moura Albino, Rafaela Fernandes Silva, Paloma Bomfim, Luiz Henrique Moraes Caetano de Camargo, Mirian Nascimento.

Quality Control Laboratory

Patrícia Dos Santos Carneiro Matheus Trovão de Queiroz, Rubia Galvão Claudio.

Development and Innovation Center

Viviane Fongaro Botosso, Soraia Attie Calil Jorge, Fabyano Bruno Leal, Renato Mancini Astray.

Scientific Development Center

Sandra Coccuzzo Sampaio Vessoni, Mauricio Cesar Ando, Guilherme Rabelo Coelho, Monique da Rocha Queiroz Lima.

University of São Paulo

School of Medicine. São Paulo, SP, Brazil

Clinical Research Center II and Research Medical Laboratory – LIM 60, Department of Infectious and Parasitic Diseases, Clinicas Hospital

Amanda Caroline Ribeiro Sales, Amanda Nazareth Lara, Angela Carvalho Freitas, Angela Naomi Atomiya, Bárbara Labella Henriques, Camila Rodrigues, Camila Sunaitis Donini, Danielle Rodrigues Alves, Elizabeth de Faria, Fábio De Rose Ghilardi, Joana Ramos Deheinzelin, Jorge Salomão Moreira, Juliana Ishimine da Silva, Karine Armond Bittencourt de Castro, Leon Capovilla, Livia Zignago Moreira dos Santos, Luara Teófilo Pignati, Luiz Gonzaga Francisco de Assis Barros D'Elia Zanella, Mariana Maria Rocha Santos de Souza, Marília Bordignon Antonio, Marjorie Marini Rapozo, Michel Silvio Duailibi, Natacha regina de Moraes Cerchiari, Patricia Rocha de Figueiredo, Pedro Henrique Fonseca Moreira de Figueiredo, Raphaella Goulart de Souza Vieira, Renata Pissuto Pinheiro, Ricardo de Paula Vasconcelos, Rosário Quiroga Ferrufino, Simone de Barros Tenore, Tatiana Fiscina de Santana, Zelinda Bartolomei Nakagawa, Elaine Cristina Bau, Lilian Ferrari, Denivalda da Silva Gomes Araújo, Gabriela de Castro Keller, Ketlin Kauane Cordeiro Santos, Rosângela Vitória Soares Silva, Beatriz Sales Mourão, Taynan Ferreira Rocha, Carlota Miranda Paredes, Carolina Cardona Siqueira Lobo, Taís Vargas Freire Martins Lúcio, Athos Nascimento Souza, Elenn Soares Ferreira, Gabriel Lopes Borba, Neivaldo Fiorin, Thiago Evaristo Tavares Luzzi, Denise Sales Mourão, Ederson Santo Xavier, Nailson de Jesus

Ramos das Virgens, Thiago Antonio do Nascimento, Bruna Samanta da Silva Moreira, Karine Milani da Silva Dias, Leandro Concolato Miranda, Mary Helen Oliveira Morais, Priscilla Almeida Souza, Rayana Silva Paes, Geovanna Guarnier Cardin Farias, Gustavo Coutinho Rezende, Rosimeire Aparecida da Silva Zabotto, Verônica dos Anjos Souza da Silva, Ana Paula da Silva Barros, Clemildes Vieira de Almeida, Gislayne Aparecida de Lima Marcelino, Jéssica Aparecida Soares, Josélia Bezerra dos Santos, Márcia Alves de São Pedro, Maria Esmelindra Monteiro de Moraes, Helena Tomoko Iwashita Tomiyama, Alberto Hiroyuki Tomiyama, Aline Tatiane Lumertz dos Anjos, Andrea Niquirilo, Claudia Satiko Tomiyama, Elisabeth Alves Pereira, Eric Silvestre, Maria Angelica Alcalá Neves, Raissa Reis Silva, Yasmine Perez Levy Ribeiro, Maria Cândida de Souza Dantas, Issler Moraes da Silva, Renan Fernandes Carvalho.

Institute of Tropical Medicine (IMT-SP)

Ester Cerdeira Sabino, Maria Cássia Mendes Correa, Anderson de Paula, Tania Regina Tozetto Mendoza, Mariana Severo, Jaqueline Goes de Jesus, Flávia Sales, Erika Manuli, Darlan da Silva Cândido, Ingra Morales.

Laboratory of Clinical and Molecular Virology, Department of Microbiology, Institute of Biomedical Science. São Paulo, SP, Brazil

Edison Luiz Durigon, Danielle Bruna Leal de Oliveira, Erika Donizette Candido, Guilherme Pereira Scagion.

Emilio Ribas Institute of Infectious Diseases, São Paulo, SP, Brazil

Luiz Carlos Pereira Junior, Ana Paula Rocha Veiga, Guilherme Assis dos Anjos, Alexandre de Almeida, Najara Ataide de Lima Nascimento, Diogenes Coelho Junior, Gabriela Prandi Caetano, Ana Carla Carvalho de Mello e Silva, Rafael Affini Martins, Natália Mercedes Cabral Amdi, Cinthya Mayumi Ozawa, Magna Magalhaes Siva, Anna Karina Queiroz Mostachio, Katia Sayama Tsutui, Ana Paula Augusto dos Santos, Alessandra Moreti dos Santos, Marcia Aparecida dos Santos Gouveia, Lilian Mathias Moreira, Léia Dias Barbosa, Margarete Rodrigues de Carvalho, Mirian Ishi, Mariana Takahashi Ferreira Costa, Nelson Alberto Freitas Guanez, Odijoselia Ferreira de Sá, Virginia Barbosa Leite, Luciana Elizabete Cunha, Ivan Máximo da Silva, Vinicius Silva Araújo, Marian Romero Soares Rodrigues, Adriano Samoel Batista de Souza Nascimento, Marco Aurélio Conceição, Catia Dionisio dos Santos, Luciana Aparecida Pereira, Alessandra de Fatima Margarida, Debora Carla dos Santos, Andréia Aparecida Hermann, Ana Kesia de Souza Lima, Nailda Dantas Nunes Leal, Sergio Luiz de Lara Campos, Mariane Pereira, Caroline Franco Zanotti, Paloma Martins Vieira, Paloma Priscila Rocha Aronca, Denis Jose Fumagali, Alpetras Martins Maciel, Thais Monteiro Nunes Pereira, Milton Tadeu da Silva, Bruno Tenório de Oliveira, Maria Elena Ana Correa da Silva, Margarete Leme, Mercia Rocha Moreira, Silvia Garcia, Vlaudeflide dos Santos, Maria Elizabete Mendes Alves dos Santos, Bianca Carolina Ferreira, Sonia Silva Ferreira, Leticia Kadiri da Silva, Neide Aparecida Leite dos Santos, Fernanda Lima Arruda, Vilma Aparecida Adami, Claudia Solange da Silva.

Instituto Israelita de Ensino e Pesquisa Albert Einstein, São Paulo, SP, Brazil

Luis Fernando Aranha Camargo, Carolina Devite Bittante, Telma Priscila Lovizio Raduan, Mariana Silva Soares, Maria Clara Pimentel Lopes, João roberto Resende Fernandes, Kamilla Ferreira de Moraes, Ariane Teixeira Vicente, Ericka Constantinov Oliveira, Aleksandra Cristina Mussi, Cristiane Okada, Bruna Camargo Gonçalves, Roberta Carolina Haddad, Caroline dos Reis Pedroso, Mariza Kogake Nocamatsu, Elke Ferreira Salim, Tarsila Gomes Feijão de Oliveira, Elaine de Jesus Santos, Bruna Camargo Gonçalves, Daniela Regina Gusmão Ferreira, Luciane dos Santos Vieira Alves, Hannah Maureen Garcia Mota, Mariana Gabriela Rodrigues, Gisele Alves Pinheiro, Luiza Blumer Ribeiro, Isabel Cristina dos Santos, Regiane Mendes Brandão, Ariana Silva de Lima Teixeira, José Carlos da Silva, Rosana Soares Gomes, Fernanda Oliveira Marcelino da Silva, Giselia Martins Dantas Silva, Aparecida Sampaio Sousa, July do Nascimento Alves, Thais Helena Costa Petrin, Daniela Boschetti, Amanda Tarratacada Guimarães, Danilo Souza Vanglerini, Juliana Alves de Lima, Letícia de Mello Soares, Julia Alves de Lima, Adriana Guilherme, Lorena Silva Brasil, Felipe Rodrigues Nogueira Silva, Diana Freire de Brito, Rebeca Cezário Freire de Araújo, Sandra Rodrigues Rocha, Lucas Soares Cardoso dos Santos, Raquel Fidelis Teixeira Bonfim da Silva, Jeane Silva Menezes, Talita Santos Martins Vaglerini, Danielle Santana de Oliveira.
